# Sample Preparation for Multi‐Omics Analysis: Considerations and Guidance for Identifying the Ideal Workflow

**DOI:** 10.1002/pmic.13983

**Published:** 2025-06-23

**Authors:** Breyer Woodland, Luke A. Farrell, Lana Brockbals, Maria Rezcallah, Aiden Brennan, Emily J. Sunnucks, Sam T. Gould, Aleksandra M. Stanczak, Matthew B. O'Rourke, Matthew P. Padula

**Affiliations:** ^1^ School of Life Sciences and Proteomics Lipidomics and Metabolomics Core Facility Faculty of Science University of Technology Sydney Ultimo New South Wales Australia; ^2^ Department of Forensic Pharmacology and Toxicology Institute of Forensic Medicine University of Zurich Zurich Switzerland; ^3^ Centre for Forensic Sciences School of Mathematical and Physical Sciences University of Technology Sydney Ultimo New South Wales Australia; ^4^ Australian Institute for Microbiology and Infection Faculty of Science University of Technology Sydney Ultimo New South Wales Australia

**Keywords:** comparative proteomics < Technology, metabolomics < Technology, multi‐omics, sample preparation < Technology, systems biology

## Abstract

Advances in methodologies and technologies over the past decade have led to an unprecedented depth of analysis of a cell's biomolecules, with entire genomes able to be sequenced in hours and up to 10,000 transcripts or ORF products (proteins) able to be quantified from a single cell. Methods for analysing individual omes are now optimised, reliable and robust but are often performed in isolation with other biomolecules considered contaminants. However, there is a growing body of systems biology studies that aim to study multiple omes from the same sample. This review details the current state of the “multi‐omics” field, trying to define what the field is, the methodologies employed and the challenges facing researchers in this field. It also critically evaluates whether these approaches are “fit‐for‐purpose” and how the field needs to evolve to enhance our understanding of how biomolecules from distinct omes interact with one another to alter cellular phenotype in response to change.

## 1 A Brief History of Multi‐Omics

The use of the suffix “ome” traces back to ∼1926 when the term “genome” entered the English language [1], while the first use of an “omics” term is reported to be 1986 when “genomics” was first used by Tom Roderick in a discussion over beers at a meeting discussing the mapping of the Human genome [2]. The terms “proteome” and “proteomics” appeared in 1994 [3], after which the creation of new ‐omes and ‐omics exploded to the thousands in existence today, some of which are not related to biology and a number of which are nonsensical. The ‐ome suffix is referring to the entirety of whatever the prefix is, for example, the “proteome” of something (cell, tissue, etc.) being the entire complement of proteins in that something. However, confusion can often be introduced through the poor definition of the something, such as the proteome of an entire organism being different to the proteome of a single cell of that organism, or what is usually defined as the proteome actually being the “ORFome” or the potential protein content as predicted from Open Reading Frame (ORF) translations of the genome, which is different to the actual proteome which consists of proteoforms. A review on these problems of nomenclature is available [4].

**FIGURE 1:**
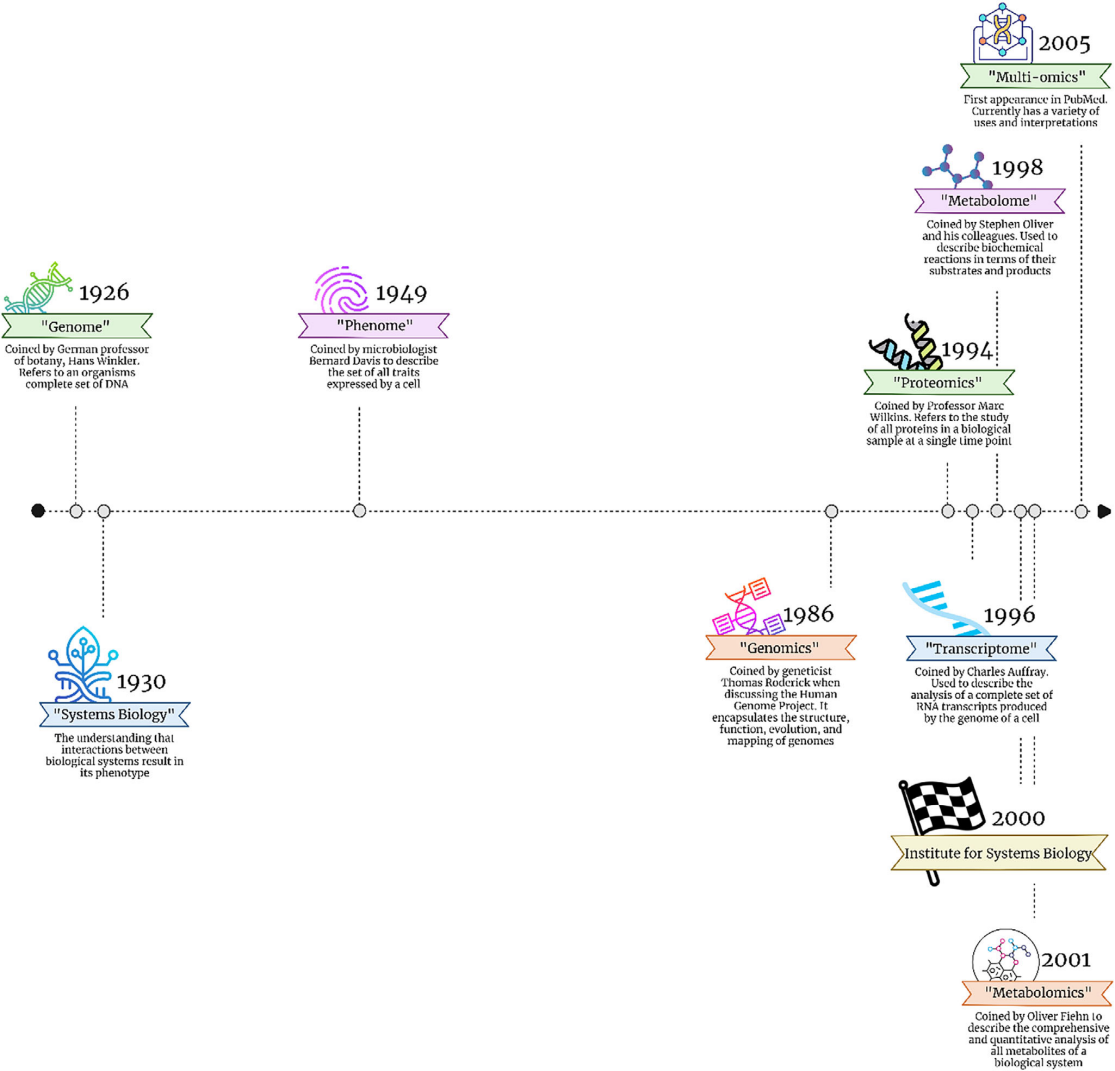
The history of the progression of different omics fields, from the earliest mention of the genome to the rise of multi‐omics.

The term “multi‐omics” first appeared in PubMed in 2005 [5], whereas the broader term “systems biology” has existed in different forms since the 1930s [6], before becoming a more formalised discipline in ∼2000 with the establishment of the Institute for Systems Biology [7]. The use of the term “multi‐omics” in preference to “systems biology” seems to be a reductionist act, restricting the scope to 2 (maybe 3) omes, such as genomics and/or transcriptomics and/or proteomics and/or metabolomics. Similarly, the term “phenome”, which is concerned with the study of phenotype was first used in 1949 [8] and is similarly misused [9]. Thus, there is a strong case that the term “systems biology” should be utilised. However, if a team is dedicated to studying genomics, transcriptomics and proteomics, to specifically examine whether genotype is being reflected in the phenotype, the question becomes whether they are a systems biology team, a phenomics team or a multi‐omics team. While this is a semantic argument, the term multi‐omics currently has a variety of uses and interpretations, and in our opinion, a naming convention should be adopted to create clarity. One such convention was defined by Krassowski et al., as being “three or more omic datasets coming from different layers of biological regulation—not necessarily within one level” [10] (see Figure 1 for a chronological history).

## 2 What is Multi‐Omics?

Multiomics, multi‐omics, integrative omics, “panomics” or “pan‐omics” is the integration of data from separate analyses of individual collections of related biomolecules (omes), ideally from the same individual sample but also from replicates of the same sample. It is essentially a field of bioinformatics, where software platforms are created that allows integration of data that has been separately collected in different experiments and tries to connect those different omes to understand the interactions that occur between components of those different omes and how they can change.

As for all experiments that aim to make quantitative measurements of a system that contains a broad range of interacting components, experimental design and appropriate sample preparation is absolutely critical. In our opinion, the single most important decision to make before commencing a multi‐omics workflow is whether to use a single sample and fractionate that into all of the different omes or to use biological replicates of a sample with each biological replicate being used to extract a specific ome. Both have their caveats, and it is the aim of this review to provide guidance to researchers about which route will best serve their specific needs. There is no universal multi‐omics sample preparation method that allows recovery of every single molecule and researchers will need to optimise their protocols for their specific biological system and for the resources they have available.

## 3 Sample Preparation Considerations for Multi‐Omics

A single biological sample can be split into its constituent DNA, RNA, protein, metabolite and lipid fractions. However, this approach often does not result in the optimum extraction of each of these fractions when compared to a sample preparation approach that focuses on isolating a single fraction and discarding the rest. When attempting to perform a multi‐omics analysis, the question then becomes whether a sample can be effectively divided up to accommodate parallel analyses or whether sufficient biological replication can overcome the effects of using what are effectively different samples as the phenotype between separate biological replicates will be subtly different. The classic example of this is the use of methylation arrays to determine levels of gene activation or silencing. It is well understood that if a gene is silenced due to hypermethylation then the resulting transcript and proteins do not get transcribed or translated respectively [11]. However, this is a temporal phenomenon and therefore gene methylation analysis, proteomics and transcriptomics should all be performed on the exact same sample. With this requirement in mind, the key consideration then becomes whether a sample harvested and stored for RNA/protein/gene methylation analysis is compatible with the subsequent workflows necessary to analyse these molecules.

One example of this is plasma, which is routinely collected for pathological testing. It has been shown previously that plasma can be safely stored at −80°C for lengthy periods of time and can also withstand multiple freeze thaw cycles and still be suitable for proteomic analysis [12]. However, the same cannot be said for mRNA which will readily degrade when subjected to freeze/thawing [13]. While the stability of most metabolites was found to be not significantly impacted by long‐term storage at –80°C, individual small molecules might be susceptible to alterations [14, 15]. Additionally, for metabolomic studies, the number of freeze‐thaw cycles should be limited to as few as possible to prevent oxidation and degradation. For plasma samples, no more than three consecutive freeze‐thaw cycles were found advisable for metabolomics studies [16], however we strongly advise that samples be aliquoted and individually stored at –80°C so that no refreezing is required. In addition, there is evidence that plasma might be a better sample choice than serum due to confounding data created by the presence of platelets [17]. Lastly, cell culture conditions and routines have also been shown to be a source of irreproducibility [18].

All of these considerations require a change in thinking when approaching a study from a multi‐omics perspective, necessitating a clear and careful plan for the order of analysis. Conversely, some sample preparation approaches for single omes can also allow for multiple analyte extractions (often with particular sample fractions intended to be discarded), meaning a sample can effectively be “reused” in order to perform multi‐omics analyses. However, these methods often only consider the ideal conditions for the extraction of a specific analyte from the whole, leaving the remaining fractions potentially being sub optimal for their own subsequent analysis due to containing reagents incompatible with analysis methods (surfactants, salts, organic solvents, etc.) or being in volumes that are difficult to concentrate. With this in mind, the next point of consideration becomes the methods used for individual ome analysis and their relative compatibility with downstream multi‐omics analysis.

## 4 Analysis Methods for Individual Omes

### 4.1 Genome

**FIGURE 2:**
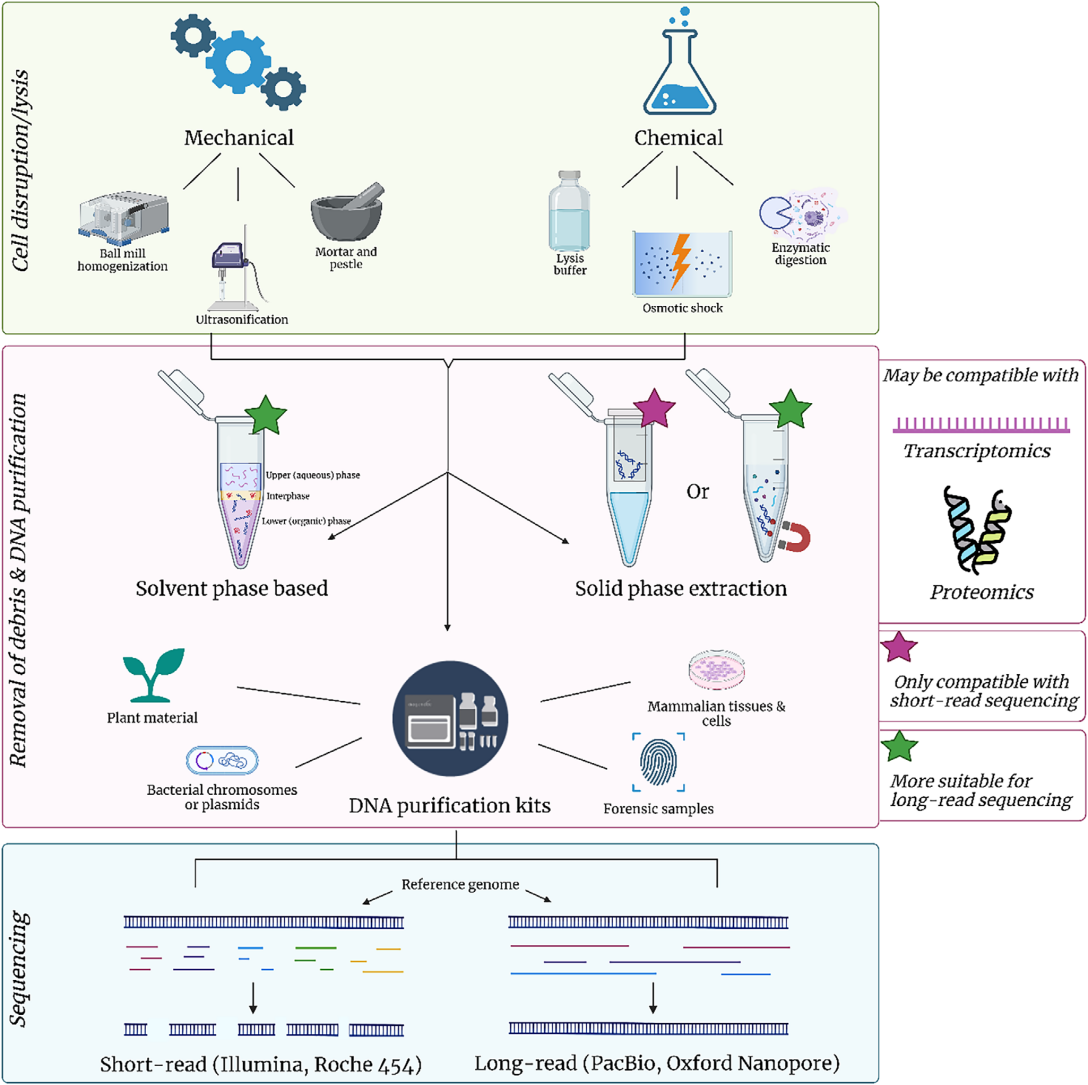
The progression of sample preparation from sample to either long read or short read sequencing.

Genomics encompasses the mapping, sequencing and analysing of an organism's genome. While the term genomics was first used in 1986, DNA sequencing technologies began to develop in the late 1970s with two pioneering approaches from Maxam & Gilbert [19] and Sanger [20]. Since then, the field of DNA sequencing evolved in its capacity, capability and applications, particularly with the completion of the first entire bacterial genome in 1995 and the first completed draft of the human genome in 2001 [21]. The advancements in genome sequencing since the 1990s can be classified into three revolutions: (1) whole‐genome shotgun sequencing (Sanger sequencing), (2) high‐throughput sequencing (short‐read sequencing), and (3) single‐molecule sequencing (long‐read sequencing) [22]. In this review, we will focus on sample preparation for the two latter revolutions: short‐read and long‐read sequencing methods (see Figure 2 for an overview of workflows).

There are numerous platforms for short‐read sequencing, however, Illumina is the most popular next generation sequencing (NGS) platform, controlling the majority of the market due to its high‐throughput and low cost for large scale screenings [23]. Short‐read methods require DNA extraction processes that result in short fragments of DNA ranging from 150 to 300 base pairs. Generally, protocols for DNA extractions include (i) cell disruption or lysis; (ii) removal of cellular debris, lipids, and proteins; and (iii) DNA purification and concentration. Cell disruption or lysis is dependent on the sample type but include both chemical and mechanical methods [24]. Chemical methods include osmotic shock, enzymatic digestion with lysozyme or proteinase K or lysis buffers such as potassium ethyl xanthogenate (XS) buffer supplemented with Tris‐HCl and 1% sodium dodecyl sulphate (SDS). Mechanical methods include homogenization with a mortar and pestle, bead beating or ultrasonication. Solvent phase‐based extractions or solid‐phase extractions (SPE) may also be employed to remove cellular debris and contaminants, such as lipids and proteins [24, 25]. Most commercially available DNA extraction kits utilise solid phase extraction methods, employing either silica‐based filter columns or various‐polymer‐coated magnetic beads. These polymers possess a strong affinity for nucleic acids, allowing them to bind reversibly upon addition of specific reagents [26]. Briefly, SPE involves the immobilization of DNA to the beads or filter, followed by washing steps with ethanol to remove contaminants (protein salts, cell residue), before elution with TE (Tris‐EDTA) buffer. Use of molecular biology grade nuclease‐free water instead of the TE buffer should be considered in cases where avoiding interference during downstream processing is priority over long‐term storage. Furthermore, reducing the volume of elution fluid can be advantageous in achieving higher DNA concentrations, particularly when the yield is expected to be low. Once DNA is eluted, purified DNA needs to be properly stored to avoid compromising the quality of the sample. Long‐term storage should be in slightly basic conditions to avoid acidic hydrolysis of DNA at –20°C to –80°C and thawed once only to avoid degradation or adsorption of nucleic acids to tube walls [27, 28, 29].

With any omics workflow, extraction methods are dependent on sample type. For example, plant material DNA extraction can be challenging due to the presence of polyphenols, polysaccharides and secondary metabolites [30]. Oxidation of such compounds and the coprecipitation of polysaccharides can interfere with extraction methods resulting in low yield and low purity of DNA. Conversely, Cetyltrimethylammonium bromide (CTAB) extractions with additives such as polyvinylpyrrolidone has been demonstrated to improve DNA extractions of plant material [31]. Commercially available extraction and purification kits are usually specialized and optimized for a particular sample type such as mammalian tissues and cells, bacterial chromosomes or plasmids, and forensic samples (blood, hair, saliva). Therefore, such kits are often preferred due to their reproducibility and potential for high‐throughput and automation [32].

Short‐read sequencing technologies have high sequence accuracy (∼0.01% error) but are not appropriate for detailed assessments of genetic context. One example is in eukaryotic and bacterial genomes that have long spans of repetitive regions where it is difficult to reconstruct the genome due to fragmentation [33]. This limitation can be overcome with long‐read sequencing using platforms such as nanopore sequencing (Oxford Nanopore Technologies (ONT), or Single Molecule‐Real Time (SMRT) sequencing (Pacific Biosciences)) [34]. In long‐read approaches, there are thousands to hundreds of thousands of bases in a single read, allowing for greater confidence of genetic context. Both SMRT and ONT have a lower read accuracy in comparison to short‐read sequencing but produce complete de novo genome assemblies which suffer from high error rates [35, 36, 37]. However, high error rate limitations, can be overcome bioinformatically with de novo hybrid assembly algorithms using both short and long‐read data [38, 39]. In the case of bacterial species, long‐read sequencing can reconstruct and fully ‘close’ chromosomes and plasmids. This is a significant advantage for surveillance studies, as it enables us to better understand the evolution and dissemination of genetic features across geographical regions and over time [40, 41].

To maximize the power of long‐read sequencing, isolating high quality, high molecular weight (HMW) DNA is extremely important, however, most cell lysis and DNA extraction protocols and kits are designed and optimized for short‐read sequencing [42]. For example, ultrasonication to lyse cells or multiple centrifugation steps using filter columns are too harsh for HMW DNA extractions as they typically shear DNA into fragments too small for long‐read sequencing [43]. This can make HMW DNA extractions challenging for tough plant material or bacteria with rigid cell walls. Generally, traditional mechanical cell disruption methods should be avoided and instead modified bead beating or grinding and nuclei isolation techniques for plant material, and enzymatic or detergent‐based chemical lysis methods for bacterial samples to increase the recovery and yield of HMW DNA should be utilised [44, 45]. Additionally, vigorous mixing by pipetting or vortexing should be minimized.

Traditionally, the “gold standard” and go‐to method for HMW DNA extraction is the phenol:chloroform:isoamyl alcohol extraction method [46, 47]. However, despite its advantages, including lower cost and the ability to produce high‐quality long DNA fragments, this method is much more time consuming, labour‐intensive and uses hazardous organic solvents [43, 48, 49]. Additionally, multiple steps require sample transfer to new tubes, increasing the risk of contamination or DNA degradation/loss. For high throughput analyses of large cohorts, commercially available kits are beneficial in ensuring minimal DNA loss and high reproducibility [44].

From a multi‐omics perspective, DNA extraction workflows often include RNAse treatment to remove contaminating RNA, thereby removing RNA as an analysable ome. However, if one is interested in both genomics and transcriptomics data, DNA and RNA can be extracted from the same sample using solvent‐based extractions such as guanidinium thiocyanate‐phenol‐chloroform [24]. This method employs acidic conditions whereby phase layers are formed in which RNA remains in the upper aqueous phase and DNA and proteins remain in the interphase or lower organic phase. RNA can then be precipitated from the aqueous phase with isopropanol and DNA can be isolated from the organic phase by ethanol or isopropanol precipitation [50].

Alternatively, commercially available “all‐in‐one” kits exist to co‐extract DNA and RNA [51] at the same time. When extracting DNA/RNA, proteins are generally considered a contaminant or ‘waste’ and are often degraded and removed using proteinase K. However, if proteinase K is not used in the extraction procedure, the leftover protein can be digested and analysed using well‐established tryptic digestion protocols in order to perform mass spectrometry‐based proteomic analysis. It should be noted that it is essential to ensure the complete removal of any residual chaotropic salts used in DNA extraction buffers to prevent inactivation of trypsin. Purification of proteins post‐DNA extraction using acetone precipitation or SP3‐based sample preparation [52] also allows for the analysis of the proteome following DNA/RNA analysis [53]. However, these methods have their own issues and caveats which will be explored later. Metabolites and Lipids are also not easily analysed from the residual fractions post DNA/RNA extraction as many organic solvents will dissolve, remove and/or modify metabolites and lipids, making them unsuitable for later analysis.

### 4.2 Transcriptome

The transcriptome was first investigated in the 1990s and is the analysis of the complete set of ribonucleic acid (RNA) transcripts produced by the genome of a cell, providing a dynamic snapshot of the total transcripts present in a cell at a specific point in time [54]. The transcriptome can be investigated to provide insights into gene expression and regulation, and the addition of transcriptomics into a multi‐omics workflow allows for a comprehensive understanding of cellular processes as the intermediary between genomics and proteomics, that is, the genotype and the phenotype. Transcriptomics aims to identify messenger RNA (mRNA), ribosomal RNA (rRNA), transfer RNA (tRNA), microRNA (miRNA) and non‐coding RNA (ncRNA), to quantify the change in expression of transcripts under varying conditions [55]. Transcriptomics is a common omic technique utilised when investigating diseases and is frequently used in combination with proteomics, providing a molecular link between genetic information and the proteome [56], although poor correlation between the two is often observed [57, 58, 59].

Initially, individual transcripts were studied via low‐throughput methods where mRNA underwent reverse transcription to convert the mRNA to complementary DNA (cDNA) using reverse transcriptase, which was subsequently analysed with Sanger sequencing, obtaining random individual transcripts called Expressed Sequence Tags (ESTs) [54]. This approach was replaced by microarrays [60] and then Real Time PCR (RT‐PCR or qPCR) that produces a quantitative measurement of a specific transcript's abundance [61]. More recently, high‐throughput methods such as total RNA sequencing (RNA‐seq) [55] have been used for RNA analysis, with these methods needing only small quantities of RNA [62]. RNA‐seq is both qualitative and quantitative, allowing for the detection of transcripts, and their isoforms and alternative splicing events by the direct sequencing of RNA [55, 62]. Various platforms, including nanopore sequencing, Pac Bio and Illumina can be used for RNA‐seq to perform long‐ and short‐read sequencing [54]. In recent years, there has been a greater focus on single cell transcriptomics and many of the points raised for transcriptomics on bulk samples also apply to single cell experiments. For a detailed review on single cell transcriptomics, the reader is directed to Arya et al. [63].

Prior to RNA sequencing, high‐quality RNA must first be isolated from the sample. Broadly, this process involves mechanically disrupting the cells, or cell lysis and RNAse inactivation using chaotropic agents and detergents, before isolation of RNA from other biomolecules [54]. Variation in sample preparation workflows have been shown to impact downstream results, highlighting the importance of considering sources of potential bias which may skew the view of the transcriptome [64, 65]. Long RNA strands are sensitive to enzymatic degradation due to cleavage with RNases from improper handling or storage conditions, and the subsequent degraded RNA will affect downstream results [54, 66]. For optimal RNA isolation, fresh samples are preferable, otherwise samples should be snap‐frozen in liquid nitrogen [54, 67].

Methods for RNA isolation are performed by affinity‐based approaches or fractionation. Affinity‐based approaches use silica‐membrane spin columns or magnetic beads for RNA isolation. Silica‐gel based columns are available as commercial kits, such as RNeasy (Qiagen), and selectively bind RNA to a silica membrane in high salt conditions, before washing impurities away, and elution of RNA from the column. Similarly, a magnetic bead‐based approach can be used for mRNA whereby the magnetic beads are coated in oligo(dT) which selectively hybridises the 3′‐terminal poly(A)‐containing tails of mRNA. The mRNA is then separated from the beads by a magnetic field [54, 68]. Fractionation approaches commonly use TRIzol, a monophasic solution of chloroform, phenol and guanidine isothiocyanate is utilised to fractionate RNA, DNA and proteins from samples [59]. In this approach, the RNA is isolated in the aqueous phase, which is then precipitated with isopropanol, and subsequently treated with DNase to digest DNA contaminants [59]. Depending on the sequencing method chosen, the isolated RNA may need to be converted into cDNA using a reverse transcription enzyme [54].

The selection of a suitable sample preparation technique is dependent on the downstream application. For sequencing using RNA‐seq, microarrays or RT‐PCR, high‐purity mRNA is needed, which often means isolation and enrichment of mRNA to the exclusion of other biomolecules, or depletion of rRNA [64]. Often, oligo(dT) beads are used, however, oligo(dT) is only selective for mRNA, which may exclude other RNAs that may be of biological relevance, and the efficiency of mRNA poly(A) selection can vary depending on mRNA tail length and deadenylation, which is influenced by the gene and mRNA age [65]. Isolated RNA or cDNA must undergo additional steps to fluorescently label and fragment before hybridisation with the microarray [54].

When considering sample preparation for a multi‐omics workflow, analyte compatibility for further downstream analysis must be considered. Often protocols used to inhibit the enzymatic degradation of RNA lead to the denaturation of proteins, subsequently affecting their downstream analysis [59, 69]. Additionally, RNA extraction utilises salts and high concentrations of chaotropic agents to bind RNA to columns or beads, which are incompatible with downstream mass spectrometry‐based omics analysis [70]. Ideally, to minimise sample heterogeneity from skewing results, the different analytes should be recovered sequentially from the same sample. Bhat el al. found that sequential extraction from a single sample provided a greater compatibility of protein and RNA in their multi‐omic workflow in comparison to a parallel workflow, and additionally a smaller amount of sample was required [71].

Several studies have successfully integrated transcriptomics into a multi‐omics workflow, with Breidenbach et al., successfully extracting mRNA, lipids and proteins from a single sample of human cells using their modified bead‐enabled accelerated monophasic multi‐omics (mBAMM) method [70]. The mBAMM method lysed cells with an n‐butanol, acetonitrile and water monophasic extraction, before RNA isolation with a RNeasy‐spin column, and paramagnetic beads for rapid on‐bead protein digestion. Overall, they found that the number of genes identified was similar to the commercial kit and, although RNA concentration was lower, library preparation was unaffected. The number of features identified in downstream ‐omics analyses was not significantly impacted by the addition of transcriptomics to the workflow. However, the effect on abundance was not presented, which is crucial when interpreting ‐omics data. Similarly, Fassbender et al. fractionated mRNA and proteins from tissue samples using TRIzol, before microarray and downstream spatial proteomics analysis using SELDI‐TOF‐MS [59], although most studies using SELDI have been found to be irreproducible [72]. Although successful in analysing mRNA and proteins from the same sample, no correlation between gene expression and proteins were identified. Additionally, they found that the proteins extracted using the TRIzol protocol differed from their previously published studies which focused on protein extraction only, possibly due to loss during extraction or variation in the samples used [59].

### 4.3 Proteome

Initially coined by Marc Wilkins in 1994 as the protein compliment of the genome, proteomics is the study of all proteins in a biological sample at a single time point under defined environmental conditions [3]. As mentioned above, confusion regarding the definition of the proteome is often due to the oversimplification of proteins as canonical amino acid sequences (i.e., the product of an open reading frame (ORF) from the genome), completely ignoring the concept of proteoforms. A proteoform is the amino acid sequence of an ORF product with its respective Post Translational Modifications (PTMs), structure and function [73]. The recognition of proteoforms as the functional species of biological systems in analytical workflows is critical, as PTMs such as phosphorylation, acetylation, glycosylation and proteolytic cleavage products cannot always be inferred through other ‘omics analyses such as genomics and transcriptomics. Furthermore, proteomics assesses the quantitative change in the abundance of canonical proteins or proteoforms in a sample, not changes to protein expression which are functions of RNA or DNA activity [4]. Reviews of various mass spectrometry‐based proteomic quantitation methods including tandem mass tags (TMT), isobaric tags for relative and absolute quantification (iTRAQ), isotope‐coded affinity tags (ICAT) and stable heavy isotope labels are available [74].

**FIGURE 3:**
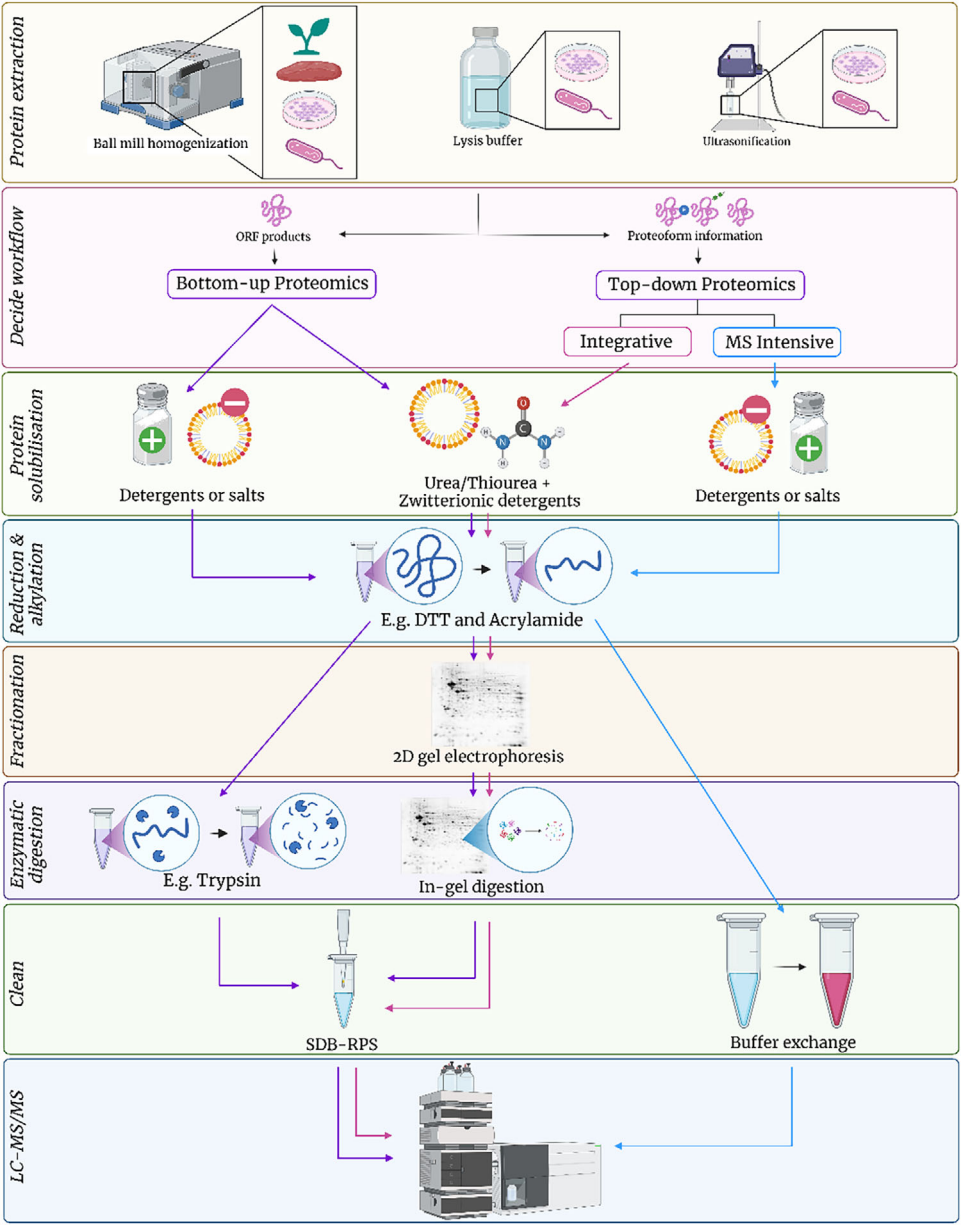
The decision tree of options available for proteomics analysis.

Proteomic analyses are multi‐faceted and can be divided into numerous sub‐categories including, but not limited to, bulk cell, single cell and cell surface proteomics (see Figure 3). Bulk cell analyses assess the changes within the proteome in a large population of cells, assuming homogeneity between cells and provides an average protein abundance profile [75]. Single cell proteomics (SCP) aims to address cell heterogeneity and diversity which cannot be assessed by bulk cell proteomic techniques. Despite the growing interest within the last decade, most proteomic workflows have been optimized for bulk cell analyses, with single cell workflows being adapted from these through volume minimisation [76, 77]. There are numerous challenges associated with SCP including sensitivity and throughput in mass spectrometry approaches and a limited number of predefined targets in antibody‐based approaches [78]. Additionally, unlike single‐cell genomics or RNA sequencing, SCP can be challenging as the proteome is highly dynamic and amino acid sequences cannot be amplified in manner similar to PCR with DNA/RNA [79]. Therefore, SCP is much more sensitive to dynamic range issues and losses during sample preparation that may compromise accurate detection and quantification [79]. Reviews on sample preparation and the associated challenges of SCP using mass spectrometry and sequencing‐based methods are available [75, 76, 80, 81] and therefore will not be covered in this review. Sample preparation methods for cell surface proteomics including surface shaving and biotinylation has also been reviewed previously [82, 83, 84] and therefore will also not be expanded upon here.

Unlike next‐generation sequencing approaches for genomics and transcriptomics, mass‐spectrometry based proteomics lacks standardised universal protocols. While some researchers in the field have pushed for a unified protocol to ensure results are robust, reliable and reproducible, there is an overwhelming number of protocols for sample preparation [52, 85, 86, 87, 88, 89]. This can make choosing a suitable method daunting and challenging. Some studies have compared particular aspects of sample preparation, but it is unlikely that there is truly a universal method that exhibits no or negligible losses or compromises. Additionally, this makes assessing all possible sample preparation protocols a futile task. It is critical to note that there is no definitive method for proteomic analysis. Rather, there is a range of techniques that are selected and employed depending on the aim of the experiment to best suit both the sample being extracted, and the downstream analysis being performed. Each method has its strengths and weaknesses, which should be recognised and addressed. As such, multiple different potential avenues should be explored in the experimental planning stage to ensure sample compatibility and effective analysis. There is a large breadth of methodologies available for analysing the proteome, but in this review, we will focus on sample preparation for mass spectrometry and affinity‐based methods.

### 4.4 Mass Spectrometry‐Based Proteomics

The majority of proteomic analyses utilise mass spectrometry as the primary analytical method. Generally, these are categorised into three approaches, “Bottom‐Up” or peptide‐centric, “Top‐Down” on intact proteoforms and less commonly, “Middle‐Down” where large peptides of 50–100 amino acids are analysed [90, 91]. In Bottom‐Up (BUP) approaches, proteoforms are enzymatically cleaved into peptides of ∼5–45 amino acids in length (<3 kDa) prior to separation by reversed phase liquid chromatography and identification via tandem mass spectrometry (LC‐MS/MS). Top‐down (TDP) approaches are often defined by methods that involve introducing intact proteins into a mass spectrometer, but here we define top‐down approaches as those that resolve, quantify and identify intact proteoforms. These approaches include both mass spectrometry‐intensive methods and two‐dimensional gel electrophoresis (2D‐PAGE) [92]. Middle‐down (MDP) approaches utilise limited proteolysis to produce larger peptides (5–30 kDa) to improve sequence coverage and improve localisation of PTMs [93].

Protein extraction and sample preparation is informed by the desired downstream processing and analysis. Therefore, before choosing a method, it is important understand the level of proteoform detail needed from a particular approach prior to sample preparation. For example, with BUP approaches, proteoform information is lost due to the protein inference problem [94]. However, this can be partially overcome using targeted PTM enrichment and N‐terminal labelling methods [95, 96, 97, 98, 99]. There are also various enrichment methods that specifically enrich for a range of PTMs such as glycosylation, phosphorylation and acetylation [100, 101, 102]. These approaches require their own bespoke methodology that specifically isolates a subset of the sample with unique chemistry and complete disregard for other analytes. It is therefore our opinion that they are incompatible with current multi‐omics workflows and instead should be performed on a fraction of the original sample prep, separate from other aspects of a multi‐omics workflow. Though the data can later be integrated with other omics datasets, this is a bioinformatics problem and beyond the scope of this review. It is our opinion that if the experiment aims to quantify and identify proteoforms rather than ORF products, a Top‐Down approach should be taken. Middle‐down approaches aim to produce larger peptides to localise PTMs to a specific location, however this has not been clearly demonstrated in the literature.

The first step of any proteomics workflow (and in general, any omics workflow), regardless of the analytical technique employed, and is establishing adequate sample collection, handling and storage prior to analyte extraction. Generally, samples should be stored at −80°C avoiding multiple freeze‐thaw cycles to preserve the integrity of proteome at the time of sample acquisition and limit possible protein degradation or induced artefactual modifications. Tissue may also be preserved using a formalin‐fixed paraffin‐embedded (FFPE) method for an alternative means of storage that is relatively inexpensive, as they can be stored at room temperature for longterm [103]. There are challenges associated with FFPE samples for proteomic analysis including the removal of paraffin wax and rehydration of embedded tissue, and the formaldehyde induced intra‐ and inter‐crosslinking of proteins during the fixation process that makes lysis and digestion more difficult. The majority of these challenges have been overcome by using clearing washes such as heptane, xylene and methanol to deparaffinize and rehydrate tissue followed by lysis and homogenization using sodium deoxycholate (SDC) and bead‐beating [104]. Other approaches also use high pressure methylene hydrolysis to cleave and remove formaldehyde crosslinks with great effect [105, 106]. With quality sample preparation it has been demonstrated that proteome and phosphoproteome of FFPE and fresh frozen tissue are highly comparable [107].

Generally, sample preparation for a basic LC‐MS/MS workflow includes the following: (1) protein extraction and solubilisation, (2) reduction and alkylation of disulphide bonds; (3) removal of ‘contaminants’ or analytes that are not of interest (i.e., nucleic acids, lipids, etc., if interested in analysing the proteome only); (4) fractionation or enrichment depending on the complexity of the sample, aim of experiment, and interest in specific modifications; (5) enzymatic digestion into peptides (BUP only); and (6) LC‐MS/MS.

Tissue disruption, cell lysis and protein extraction methods designed to maximize overall protein yield varies with sample type. Proteomic analyses have been applied to a broad range of sample types but here we will focus on sample preparation for tissue, plant material, bacterial cells and blood (plasma and serum). Sample preparation guidelines and protocols for other sample types such as bone and biofluids (i.e., urine, cerebrospinal fluid and saliva) are available [108, 109, 110, 111]. Broadly, these extraction methods include physical disruption or homogenization, and reagent‐based methods such as detergents/surfactants, chaotropes and/or salts. For tissue [112] and plant material [113, 114], mechanical homogenization methods using bead beating techniques followed by solubilisation in protein extraction buffers containing detergents and chaotropes have been demonstrated to improve protein extract yields compared to manual grinding with a mortar and pestle [112].

In these approaches, the frozen sample is agitated vigorously in a pre‐cooled tube containing metal, glass or ceramic beads to produce a finely pulverized powder with increases surface area for protein accessibility and solubility. Cells from bacterial and mammalian cell culture are washed with PBS to remove growth media and Foetal Bovine Serum proteoforms followed by protein extraction using lysis buffers such as 1%–4% SDC or 1% sodium dodecyl sulphate (SDS) paired with a buffer such as Tris‐hydrochloride or 2‐[4‐(2‐hydroxyethyl)piperazine‐1‐yl]ethanesulfonic acid (HEPES). In bacterial preparations, homogenization via ultrasonication or bead mill techniques and the addition of lithium chloride can also be utilized to improve extraction efficiency by further disrupting rigid bacterial cell walls, shearing DNA, and removing protein associated capsular material. If performing fractionation using 2D‐PAGE, the sample should be filtered with 300 kDa centrifugal filtration devices to further remove cell wall contaminants that will interfere with isoelectric focussing [115]. Due to the large dynamic range of plasma and serum, depletion or fractionation methods can be employed to remove high abundance species prior to 2DE or LC‐MS/MS. One must be aware of the limitations of depletion kits, as co‐depletion may occur during the removal of high abundance species such as albumin [116, 117, 118]. Other methodologies such as Volumetric Absorptive MicroSampling (VAMS [119]) allow for deeper proteomics analysis, however, the nature of these methods remove everything except for the proteome and attempts have not been made to integrate these devices into multi‐omics workflows.

To preserve the integrity of the proteome, extraction and lysis buffers should be supplemented with broad spectrum protease inhibitors. This ensures that any PTMs detected will be either a known artefact of the sample preparation, such as oxidation of methionine residues or deamidation of glutamine and asparginine [120, 121, 122], or a true reflection of biology. Often researchers will forego the use of protease inhibitors if they are using “harsh” extraction protocols, such as boiling samples in 4% SDC. However, this assumes that all proteases are denatured by heat and high concentrations of detergents, disregarding the possible changes and artefacts to the proteome induced by exposure to high temperatures. In addition, the addition of inhibitors introduces more small molecules into the sample which will become part of the metabolite fraction of multi‐omics is pursued, although there are no reports of the effect on metabolome analysis.

In most proteomic workflows, disulphide bonds between sulfhydryl groups of cysteine side chains are reduced and alkylated. Numerous works has been performed to compare the efficacy of various reducing and alkylating agents [123, 124, 125, 126, 127]. Most commonly, dithiothreitol (DTT), tributylphosphine (TBP) and tris(2‐carboxyethyl)phosphine (TCEP) are used for reduction, and acrylamide monomers, iodoacetamide and chloroacetamide are used for alkylation. Reduction and alkylation methods should be optimised with adequate concentrations and choices of reagents to ensure complete reduction and minimise side reactions. Unwanted side reactions on amino acid residues other than cysteine have been observed using iodine‐containing alkylating reagents resulting in artefactual modifications [128]. For example, the use of iodoacetamide may cause the formation of carbamidomethylated and carboxymethylated methionine side chains. Unwanted side reactions appear to occur less with acrylamide [124].

Regardless of sample type, lysis and/or extraction buffer compatibility must be considered carefully as the use of certain reagents may limit the ability to perform, or increase the difficulty of, downstream workflows. For example, proteins extracted using charged surfactants such as SDS or salts create problems for isoelectric focusing for 2D‐PAGE. When using 2D‐PAGE fractionation, samples should be extracted using 7 M urea and 2 M thiourea paired with a zwitterionic detergents such as 1% C7BzO or 4% CHAPS. Additionally, some extraction buffers with high salt concentrations simply are not compatible with LC‐MS/MS due to their ability to contaminate chromatography columns and suppress mass spectrometry signal [89]. Further, organic solvent or acid precipitation can be used to precipitate and pellet the protein fraction through centrifugation, allowing contaminants, interfering reagents and unwanted biomolecules to be removed in the organic solvent/acid fraction. Acetone is commonly used; however, it has been demonstrated that low molecular weight proteins (<25 kDa) can be lost in the soluble fraction. Acidified acetonitrile has been demonstrated to precipitate most proteins across a wide molecular weight range [129].

To allow for a more comprehensive proteome analysis, samples can be fractionated with a myriad of methods that can be performed online or offline. “Online” refers to fractionation methods that can directly elute into instrumentation for analysis, such as the use of a liquid chromatography setup eluting directly into a mass spectrometer. “Offline” refers to fractionation methods that are not coupled directly to analysis instrumentation with fractions collected into tubes before the next separation or analysis step. In more complex setups, multiple fractionation methods are coupled together to resolve species in multiple different dimensions exploiting different chemical properties (charge, size, hydrophobicity, etc.). Online chromatography methods are applicable for TDP and BUP, pre‐ and post‐enzymatic cleavage (integrated online), to separate intact proteins and peptides, respectively. It should be noted that all online methods can be converted to offline methods with the use of a sample collection setup, however, this requires more user input and interaction offsetting the often complicated multi‐dimensional online setups that often require specialised and expensive instrumentation. There are a wide range of online and offline setups that can be employed to fractionate both intact proteins and peptides including a myriad of liquid chromatography techniques such as reverse phase, size exclusion, hydrophobic interaction and ion exchange chromatography [130, 131, 132, 133, 134]. Alternatively, other methods non chromatographic fractionation methods exist, such as capillary electrophoresis, antibody or nanoparticle‐based, and gel‐based fractionation such as 2D‐PAGE and SDS‐PAGE [130, 135, 136, 137, 138].

For BUP and MDP workflows, after extraction and any desired offline fractionation, proteoforms are enzymatically cleaved into peptides. Trypsin is overwhelmingly used for BUP workflows due to its high cleavage specificity and production of “mass spec friendly” 2+ and 3+ charged peptides that fragment well and are well suited for reversed phase chromatographic separation [139]. However, there are a wide range of enzymes that can be used alone or in combination with trypsin such as LysC or GluC for MDP approaches [93]. Key differences between them include their cleavage specificity, and pH and temperature requirements for optimal activity.

For analysis of peptides or intact proteoforms, common contaminants that need to be removed prior to LC‐MS/MS injection include salts, lipids and remaining extraction reagents such as surfactants. In a general workflow, these contaminants can be removed at two stages, with many methods opting to remove contaminants as either part of the protein extraction method (i.e., acid/organic solvent precipitation as described above), or as a “clean‐up” step prior to LC‐MS/MS injection as favoured in “single pot” methodologies that avoid removing liquid from the sample tube. As part of the extraction process, single‐pot, solid‐phase‐enhanced sample preparation (SP3) bead‐based sample clean‐up can be employed to selectively bind protein, allowing contaminants to be washed away from the sample in a single step [52]. Post digestion, the most common form of sample clean‐up involves the acidification of peptides in an acidic loading buffer, followed by the flowing through a STAGE tip made from a Teflon disc derivatised with C8 or C18 reversed phase material or styrenedivinylbenzene‐ reverse phase sulfonate (SDB‐RPS) cation‐exchange material [140]. In the case of SDB‐RPS, acidified positively charged peptides are bound to the disc, allowing neutral or negatively charged contaminants to be washed away through a multi‐step procedure before elution with an alkaline, ammonia‐based solvent. Another key reason to employ STAGE tipping is that it can also be used as a normalisation step. The discs are able to be cut to a reproducible size that will only bind a specified amount of total peptide. This allows the STAGE tips to be saturated to the same quantity, ensuring that the final eluted sample is normalised prior to injection into the mass spectrometer.

### 4.5 Compatibility of Proteomics Sample Preparation Techniques With Other Omics Analysis

Traditional and optimised proteomics workflows are noted as being largely incompatible with a multi‐omics workflow due to analytes from other omes being considered contaminants. Reagents such as Benzonase are often used to cleave DNA precluding genomic analysis. Likewise solvent precipitation methods and STAGE tipping are used to remove lipids and metabolites, although attempts at their recovery from the unbound flow through fraction have not been reported. As such the general approach for extracting all of the omes from a sample is to split the sample as early as possible and perform optimised protocols for each required omes to extract the analytes.

There is a range of workflows attempting to extract and separate multiple omes in a one pot method [141, 142, 143, 144]. These methods generally employ the use of a solvent phase separation to separate the protein fraction (precipitate) from the metabolites (aqueous) and the lipids (organic), or some form of protein binding such as SP3 or tagged proteins binding to a specifically engineered agarose bead to reversibly bind to proteins. It is important to note, that even in optimised proteomics sample preparation, the usage of different lysis buffers and extraction solvents will result in significant changes in downstream proteome coverage, peptides matched and variability in protein concentration and recovery [145, 146]. With these multi‐omics sample preparation techniques generally employing the use of common metabolite/lipid phase separation workflows such as the Folch/Bligh‐Dyer, or Matyash method, it is unlikely that the proteome is not compromised in some capacity. Despite this, no comparison of the two methods has been published.

### 4.6 Affinity‐Based Approaches

There are numerous types of affinity‐based methods but here we will focus on currently popularized approaches, Olink and Somascan. However, the considerations and limitations of these platforms are the same for all affinity‐based methods. These methods are only able to analyse proteoforms that are soluble in buffers such as PBS or Tris‐HCl, and so forth. Surfactants, chaotropes or solvents that denature the proteoforms will interfere with the binding of the target to the antibody or aptamer. Thus, the sample preparation is normally very minimal (dilution and centrifugation), which may seem like a positive but presents as a significant disadvantage when trying to detect proteins that are not soluble in these mild buffers, providing challenges for extracting a complete proteome from more resistant samples such as plants, tissue or bacteria. New sample preparation approaches that make these affinity methods compatible with difficult samples have not yet been developed, and with the given complexity and expense of these techniques, there is unlikely to be an incentive for the manufacturers to do so.

Affinity based approaches, such as the antibody‐based Olink technology, and the aptamer‐based Somascan, have been presented as alternatives to mass spectrometry‐based approaches. Olink is an immunoassay that employs the use of paired antibodies to confirm the presence of a target protein [147] and has been shown to be capable of detecting over 5400 proteins using only 2 uL of plasma or serum, and capable of detecting proteins within a large dynamic range of fg/mL to mg/mL in quantity (https://olink.com/products/compare). Somascan is an aptamer‐based detection method that employs the use of Slow Off rate Modified Aptamer reagents (SOMAmers) to bind and detect proteins [148]. It is advertised as capable of detecting up to 11,000 proteins from a single 55 uL sample, with a dynamic range of detection in the femtomolar to micromolar concentration range (https://somalogic.com/dynamic‐range/). Olink and SomaScan have been reported in numerous studies to provide inaccurate or inconsistent results when compared with other affinity‐based methods (such as each other) and traditional mass spectrometry [118, 149, 150, 151, 152, 153]. This has been theorised by many to be due to the inherent bias of affinity/antibody‐based proteomics, with changes in protein modification, conformation or mutation being capable of producing false negative [153, 154, 155, 156, 157]. Similarly, antibody/aptamer‐based validation has the potential to provide a false positive, with the possibility that the antibody is not specific for only one proteoform [154, 155]. Issues with antibody‐based proteomics are well documented, with common problems such as cross reactivity, variability and incorrect application, and antibody quality being highly variable, with significant changes being reported between batches of the same antibodies [158, 159, 160, 161]. Another key disadvantage is the inability for antibody/aptamer‐based studies to provide proteoform level data (unless the antibody is specific for a single modification, which has been thoroughly characterised) with presence, absence (defined as below the limit of detection) and quantification being the only available metrics, thus being unable to report protein modifications or cleavage products [162]. Furthermore, antibody/aptamer‐based approaches are limited by their ability to only provide data for the proteins they are designed to bind to. In contrast, mass spectrometry‐based approaches can characterise a protein not found in a database through software based de novo sequencing [163].

Despite these limitations, the popularity of antibody‐based approaches is increasing, largely due to their ability to provide proteomic data for relatively low abundant proteins that are not easily detectible through mass spectrometry. It is our opinion that antibody and affinity‐based techniques should remain complementary to mass spectrometry, with critical findings, if not detected with mass spectrometry, validated through alternative methods. From a multi‐omics perspective, affinity‐based approaches could potentially be used to separate the omes from the unbound fraction after capture of the proteoforms, but it is likely that some biomolecules are going to adhere to the capture reagents in a non‐specific manner and be lost. There are no reports attempting to use these reagents as a component of a multi‐omics workflow.

### 4.7 Metabolome/Lipidome

As coined by Fiehn in 2001 [164], the term metabolomics defines the comprehensive and quantitative analysis of all of the metabolites of a biological system under study. Generally, all small, low molecular weight analytes (<1000 Da or < 1500 Da) are included in this definition, which also encompasses lipid molecular species [165]. Hence, lipidomics is often regarded as a subdivision of metabolomics, although lipids are chemically distinct non‐polar, hydrophobic molecules that often require a dedicated sample preparation and analysis strategy as detailed below [166]. NMR spectroscopy, GC‐MS and LC‐MS/MS are all frequently used analytical techniques for metabolomic and lipidomic studies [166, 167]. Advantages and disadvantages of NMR versus MS‐based workflows for metabolomics application have been reviewed elsewhere and will not be further extend on here [168, 169]. While it is important to emphasize that no single analytical platform can detect, identify and quantify the entirety of metabolites present in a biological system, LC‐MS/MS has become the technique of choice in recent years for many metabolomics applications due to the simplicity of sample preparation (no need for derivatization), instrument sensitivities, robustness and the ability to cover a wide range of molecule's polarities [166, 170, 171]. The latter can be achieved due to the availability of a variety of column chemistries. Complementing traditional RP columns (e.g., C18‐based) that are powerful in separating semi‐polar and/or hydrophobic metabolites (e.g., phenolic acids, flavonoids, steroids but also lipids) with hydrophilic liquid interaction chromatography (HILIC) columns is often described to increase the separation and hence detectability of polar metabolites (e.g., amino acids and derivatives, sugars and vitamins) [171, 172]. Individual applications also utilize mixed mode column chemistries, attempting to separate all metabolites in one analytical run to minimize the number of injections per sample, decreasing the run‐time or enhancing the detectability of sugars and carboxylic acids [144, 173]. Independent of the chromatography, positive and negative ionization are often used complementary to each other. An extreme case of direct‐injection‐MS (coined simultaneous multi‐omics analysis by direct infusion mass spectrometry—SMAD‐MS) has also recently been developed by Jiang et al., utilizing ion mobility separation for the simultaneous quantification of more than 9000 metabolite m/z features (polar metabolites and lipids) and 1300 proteins in less than 5 min without the use of a chromatographic system [174]. Despite this rapid MS analysis, sample preparation/extraction/fractionation is also needed for this approach, with different omes manipulated separately and then recombined for bioinformatic analysis [174].

Particularly for metabolomics studies, one must be aware that an extraction bias for certain compounds/compound classes can always occur, depending on the used extraction solvent(s) and sample preparation workflow. Previous studies showed that the same set of samples, extracted with different extraction workflows/solvents can lead to significant differences in measured metabolite levels, even resulting in contradicting biological interpretation of metabolite pathways [175, 176]. Taking this into consideration, it is recommended that for untargeted exploratory studies, an extraction method should be chosen that is as unselective and reproducible as possible. However, extraction bias can most likely never be excluded. Therefore, it is crucial to perform extensive method evaluation prior to the analysis of authentic samples to be aware of the limitations of one's sample preparation workflow.

**FIGURE 4:**
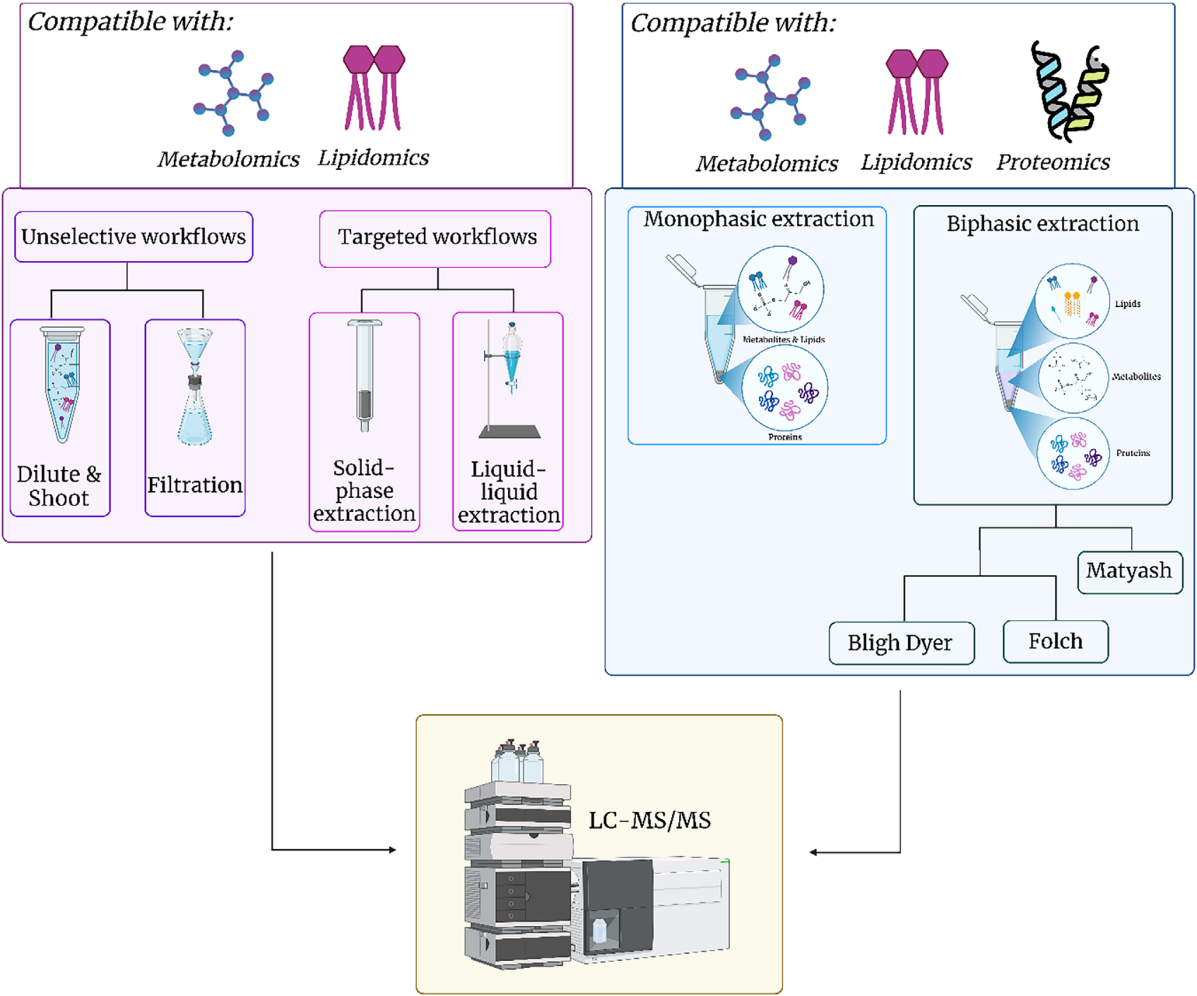
Approaches for Lipidomics/metabolomics demonstrating compatibility with other ome's.

In general, sample preparation procedures for metabolomics studies targeting polar and/or semi‐polar metabolites are often based on protein precipitation (monophasic extraction solvents) or dilute‐and‐shoot/filtration workflows (see Figure 4), which are simple extraction techniques that are as unselective as possible. More targeted extraction techniques like solid‐phase‐ or liquid‐liquid extraction (SPE or LLE) are commonly only utilized for targeted metabolomics studies, where the extraction procedure can be specifically optimized for the target metabolites or the metabolite classes under consideration [166]. More complex biphasic solvent mixtures are primarily used if a dedicated lipid analysis is also aimed for. In these cases, solvent systems like adaptations of the Bligh‐Dyer/Folch or Matyash‐extraction methods are commonly used [177, 178, 179]. Here, mixtures of chloroform, methanol and water or methyl tert‐butyl‐ether, methanol and water, respectively, are used to achieve a phase separation between a lipid‐rich organic top layer and an aqueous layer accumulating the majority of polar metabolites. Proteins are then left precipitated in a pellet on the bottom of the tube. For pure metabolomics/lipidomics studies, this pellet is discarded. For maximal reproducibility and method robustness, the aim is to have as few macromolecules (like proteins) and matrix constituents in the liquid phases as possible. This can drastically decrease matrix effects and in turn increase column lifetime [180]. When looking at the implementation of multi‐omics workflows, however, the usually discarded protein precipitate can be utilized for proteomics analysis. While historically, only specialized protein extraction methods have been considered to produce reliable proteomics data, a protein precipitate is nowadays one of the most common sources of sample material for bottom‐up and top‐down LC‐MS/MS‐based proteomic workflows [87, 181, 182]. Following this, biphasic extraction procedures are well suited for the integration of metabolomics, lipidomics and proteomics analyses. Unfortunately, such biphasic extraction protocols require careful pipetting during phase separation, which makes them low throughput and susceptible to sample loss [183]. Consequently, attempts have been made to develop monophasic extraction workflows with comparable depth, quantitative reproducibility and recovery of biomolecules as biphasic extractions, with the potential to be automated. An example of this is the bead‐enabled accelerated monophasic multi‐omics (BAMM) method developed by Muehlbauer et al. [183], which is based on an n‐butanol‐containing extraction solvent mixture with acetonitrile and water. They produced metabolomic, lipidomic and proteomic data comparable in coverage to the biphasic extraction adapted from Matyash [179, 183]. In accordance, a large comparative study by Brockbals et al. found monophasic extraction solvent mixtures to show better overall results compared to biphasic solvent mixtures, when looking at the comprehensiveness of metabolomics, lipidomics and proteomics datasets from a single postmortem tissue sample [144].

Overall, commonly utilized metabolomics and lipidomics sample preparation workflows including monophasic or biphasic extraction solvents can be recommended for multi‐omics integration, easily facilitating additional proteomics analysis from the formed precipitate. However, thorough analytical method evaluation and benchmarking of the used workflow prior to its use is crucial. Besides reproducibility of replicate extractions over several days, extraction, autosampler and freeze‐thaw stability should be tested. Proteomic extracts are generally regarded as quite stable, as a desalting/clean‐up step during sample preparation is usually followed by evaporation (e.g., under a nitrogen stream or using vacuum centrifugation). At this point, dry extracts can conveniently be stored/frozen and only re‐constituted immediately prior to mass spectrometric analysis. In metabolomics and lipidomics studies, an extract‐dry‐down‐step is often circumvented to reduce the introduction of unwanted sample preparation variability. Particularly for monophasic extractions this, however, means that the injected sample is stored in an organic solvent, which might be prone to evaporation in between multiple injection replicates (after piercing the sealed lid of an autosampler vial). Following this, the use of individual vials for repeated injections is recommended for critical, highly volatile extraction solvents [144]. In terms of autosampler‐stability, studies suggest that, for example the urinary metabolome, is not significantly altered during storage in the autosampler for 48 h at 4°C; longer extract storage times should be prevented for all matrix extracts [184, 185, 186].

### 4.8 Glycomics and Glycoproteomics

The glycome is arguably the most difficult of the ‐omes to characterise due to the huge class diversity. From a multi‐omics perspective, deep analysis of the glycome has been enabled by techniques such as Same Sample Sequential Multi‐Glycomics (SSSMuG [187]), allowing analysis of multiple glycan classes such as N‐ and O‐linked glycans, glycosaminoglycans, glycosphingolipids and polysialic acids, while also allowing analysis of the proteome at a peptide‐centric level. It is interesting to note that proteome coverage and signal intensity for the peptides analysed at the end of the SSSMuG workflow is significantly higher than the unprocessed sample, which is likely due to non‐glycosylated peptides ionising with greater efficiency [188]. While not attempted in the work detailed in the article, there is no technical limitation to performing this workflow after extraction of the metabolome and/or lipidome, allowing an unparalleled multi‐omics analysis.

### 4.9 Volatilome

Volatilomics is a sub‐field of metabolomics that analyses the dynamic, complex mix of volatile organic compounds (VOCs) that are released through metabolic processes occurring within an organism. These are compounds of low molecular weight and high vapour pressure that can be analysed collectively as the volatilome or individually as biomarkers. Volatilomics is currently utilised to identify biomarkers for specific diseases and cancers [189], monitor environmental changes [190], and explore microbial changes [191].

Non‐invasive sample matrices such as breath, saliva, sweat, faeces, and urine are often examined, and changes in VOC concentration within these samples mirror metabolic processes [189, 192]. Sample preparation techniques used with other ‐omics techniques, such as SPE or liquid extraction, are incompatible with gas chromatography (GC), and often lead to loss of VOCs through incomplete extraction, solvent interference, or a solvent delay [193]. Therefore, volatilomics typically employs a solvent‐free preparation technique such as headspace (HS) sampling, in conjunction with active or passive sampling, and is analysed in parallel with other omics techniques [190, 194]. Headspace sampling is non‐destructive, resulting in minimal sample loss and no interference with further downstream omics analysis.

The solvent‐free preparation of volatilomics utilises electrochemical sensing or analytical instrument‐based approaches with solid‐phase microextraction (SPME) or sorbent tubes, otherwise known as thermal desorption (TD) tubes [192]. A detailed review on the different VOC collection types is available [195]. Briefly, SPME consists of a fibre, typically coated with a silica stationary phase, which allows volatile and semi‐volatile compounds to adsorb based on their affinity to the stationary phase. Sorbent or TD tubes are stainless steel tubes packed with a carbon‐based sorbent material, which readily adsorbs compounds based on their affinity for the sorbent material [195]. The sorbent material varies in polarity, pore size, and volume and can be varied based on the target VOCs. Sorbent types can often skew results since compounds selectively adsorb based on their affinity for the sorbent material. In oversaturated samples, compounds with a higher affinity will displace those with lower affinity for the sorbent material causing biased analyte loss. Samples collected using SPME or sorbent tubes are subsequently analysed with one‐ or two‐dimensional gas chromatography via direct injection and desorption of the SPME fibre or a coupled TD unit. Currently, volatilomics studies are not standardised, and apply an array of different sorbent materials, sampling parameters and experimental designs, making it difficult to ascertain the best method for the collection of VOCs for different purposes and comparison. Since volatilomics is conducted in parallel with other omics techniques, normalisation of the volatilome against other omes needs to be considered. Although still a relatively new technique, volatilomics has been successfully integrated with other omics techniques such as transcriptomics, metabolomics [190] and lipidomics [196], providing additional information on biological pathways.

### 4.10 Interactomics

**FIGURE 5:**
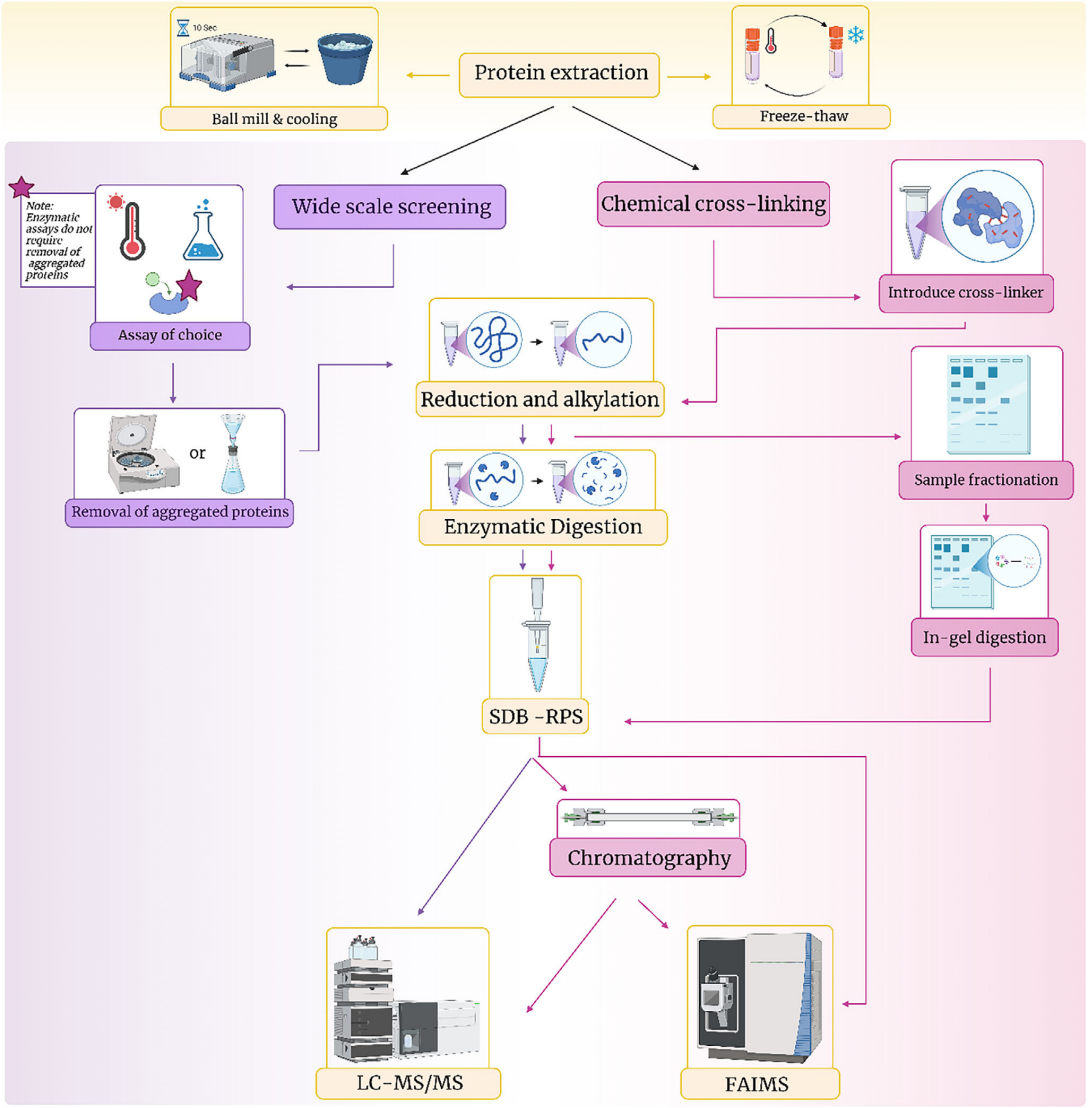
Approaches used for interactomic analysis, including crosslinking and non‐crosslinking approaches.

Native state analysis, structural proteomics and interactomics are distinct but overlapping types of omics‐based analysis. Native state mass spectrometry and structural proteomics seek to analyse biomolecules in as close to their native state as possible, involving several techniques, including chemical crosslinking (XL‐MS), cryogenic‐electron microscopy and Limited Proteolysis (LiP‐MS) [197, 198]. In contrast, interactomics, first established in 1999 [199], focuses on quantifying the interactions between biological molecules using techniques such as thermal proteome profiling (TPP) and chemical crosslinking (XL‐MS) [200, 201]. It should be emphasised at this point the overlap between these fields. One example is XL‐MS, which can provide information on the interactions between proteins and other molecules as well as the structure of protein complexes and is primarily done under native conditions. This is also true for a number of methods in these fields, and optimal workflows should therefore aim to capture interactomic and structural level information of the proteins under native conditions, minimising the effect of the workflow on the analysed molecules (see Figure 5). Many of the considerations described for affinity‐based analysis techniques also apply to interactomics.

As both XL‐MS and TPP require maintaining either or both native conformation and interactions, considerations must be made regarding changes to a number of the steps of the already‐discussed multi‐omic methodologies [202, 203, 204, 205]. During the cell lysis step, many commonly used methods, such as sonication [206], or chemicals, such as detergents, surfactants or enzymes, can break apart complexes, disrupt interactions or denature proteins. Additionally, mechanical methods such as bead milling may cause increases in temperature due to shearing forces which may also result in protein denaturation [207]. Therefore, it is important to carefully select extraction methods for these workflows. Extraction methods for interactomics and native state workflows primarily rely on using native lysis buffers in conjunction with cell membrane disruption through short bursts of mechanical disruption, interspersed with cooling or standalone freeze‐thaw cycles, to lyse cells with minimal disruptions to the molecular interactions.

Once the sample is lysed, it is imperative that the extract be stored under non‐denaturing conditions to ensure proteoforms remain in their native state and/or with intact interactions, depending on the workflow. Low concentrations of mild surfactants, such as SDC and CHAPS, or non‐ionic surfactants, such as NP‐40 and Triton X‐100, can assist with protein extraction and solubilisation. However, care must be taken to limit their concentration to limit the breakdown of interactions and the denaturing of proteoforms. Several denaturant based methods, such as TPP [208] and SPROX [209], require the protein to aggregate and be removed from the solution as a result of applying a denaturant perturbation. In these methods, different surfactants may change the denaturant curve of the proteoforms and as a result effect their solubility and later analysis [210].

The non‐ionic surfactant NP‐40 has been used in thermal proteome profiling workflows, showing minimal effect on the denaturant profiles of proteins while improving membrane protein extraction [203]. Recent work, however, presents conflicting evidence showing that non‐ionic surfactants, including NP‐40, affect the membrane proteins' thermal stability in a thermal proteome profiling workflow [211]. It is most likely impossible to completely prevent stability changes resulting from extraction and sample buffers. Therefore, buffers should be carefully selected and tested before being applied to interactomics workflows. In addition to buffer considerations, protease and phosphatase inhibitors can be used to prevent the disruption of unstable interactions or the denaturing of highly susceptible proteoforms, allowing these potentially critical molecules to be analysed.

In addition to denaturant‐based methodologies, proteolysis‐based analysis methods, such as limited proteolysis mass spectrometry Lip‐MS, are also best performed under native extraction conditions, according to Schopper et al. [204]. However, in that study, not all sample types were tested, and in the case of bacteria the extraction was performed on *Escherichia coli*, a gram‐negative bacterium, and different processes would need to be considered in the case of gram‐positive bacteria. Similar considerations would need to be made with other sample types, such as plants to overcome the difficulty in breaking apart the thick cell wall without disrupting interactions. Recently a methodology was implemented utilising Lip‐MS in plants to analyse protein‐metabolite interactions [212].

Another of the primary methods used to analyse proteoform–proteoform interactions is chemical crosslinking mass spectrometry (XL‐MS or CL‐MS). Detailed reviews are available describing crosslinkers and their respective uses [213, 214]. Within XL‐MS sample preparation, considerations need to be given to maintain conditions that prevent the breakdown of interactions and provide optimal reaction conditions for the crosslinking. Inert reaction buffers such as HEPES and PBS must be used in workflows that link amines, sulfhydryl and carboxylic acids as they do not compete for reactive sites or cause breakdown of interactions while having a neutral pH, which is required for the most efficient reaction conditions for a majority of crosslinkers. When crosslinking proteoforms, an optimal ratio of crosslinker to protein is necessary to ensure efficient crosslinking and reduce off target, non‐specific linkages. However, this can lead to detection issues in later mass spectrometry analysis, compounding upon long‐standing dynamic range issues within mass spectrometry analysis, where peptides from more abundant proteins mask the extremely low‐abundant crosslinked peptides [215, 216]. Despite this, mass spectrometry technologies such as high‐field asymmetric waveform ion mobility spectrometry (FAIMS) have been used to overcome this issue. Additionally, enriching crosslinked peptides through techniques such as strong cation exchange (SCX) and size exclusion chromatography (SEC) provides a similar improvement to subsequent mass spectrometry detection, as does proteoform separation via SDS–PAGE [213, 215, 216, 217, 218].

**FIGURE 6:**
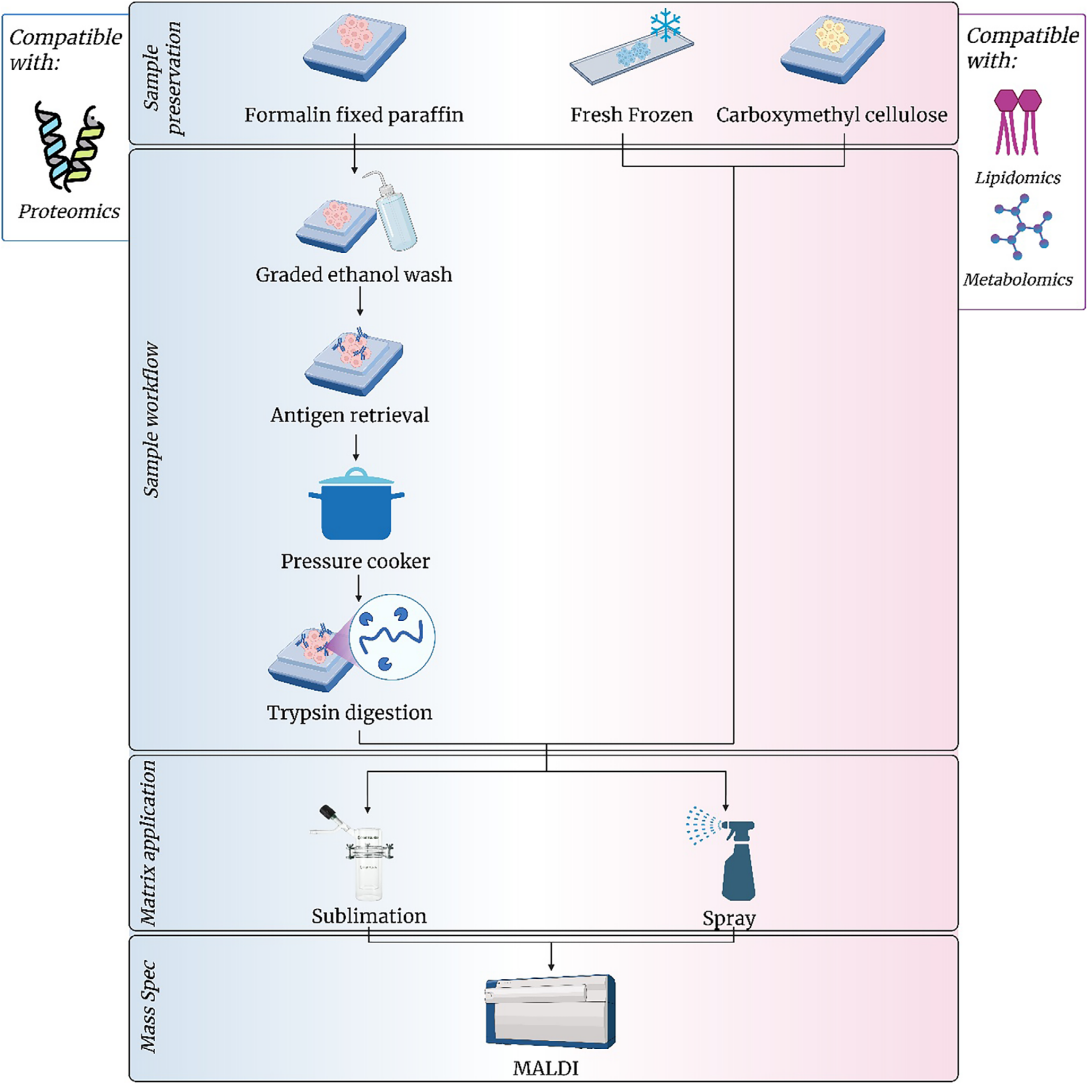
Approaches for Spatial imaging demonstrating compatibility with other ome's.

Regarding these methods use in multi‐omics workflows, TPP and other denaturant based methods have been used in a “multi‐omics” approach to measure the effect of proteoforms' interactions with metabolites or glycans, and the subsequent of effect of these small molecules on protein thermal stability or resistance to unfolding. This was used to determine protein glycosylation or interactions with metabolites, such as in the formation of efflux pumps effects, which have been shown to affect the thermostability of proteins [219, 220]. Similar approaches such as Lip‐MS have been applied to measure metabolite–protein interactions [212]. Additionally, a growing amount of research is being performed using different families of chemical crosslinkers that can be applied to analyse interactions between analytes across different omes allowing researchers to investigate a broader range of potentially linked targets. These include links between protein and glycans or proteins and lipids largely through the use of synthesized photoactivable crosslinkers [221, 222, 223]. In the case of lipid‐based XL‐MS, as in the case of denaturant‐based methods, these methods do not analyse both omes simultaneously. Rather, they identify the potential lipid binding site on the protein and therefore provide only limited information on the lipid itself. While not strictly a multi‐omics approach, such an approach still provides information regarding the interactions of different biological molecules across different levels of biological organisation. Such an approach could potentially allow for the co‐analysis of biological molecules through their interactions without sample separation or the need for tandem workflows, though more work is needed in this area (Figure 6).

### 4.11 Spatial Multi‐Omics

Spatial techniques have been employed throughout history to couple physiological observations to tissue structure and then differentiate these across cells where they would otherwise be averaged across the sample. Haematoxylin and eosin (H&E) staining (1863) [224] and antibody fluorescence (1941) [225] are some of the first examples of techniques designed to aid physiological analysis through a microscope. H&E staining is used to spatially represent cellular organelles, where fluorescence was first utilised in this context to highlight pneumococcal antigens in infected tissue.

In the present day, the frontier of science has advanced to the level of molecular analysis with new techniques and protocols catered to molecule‐specific pathways; including genomics (genes), transcriptomics (mRNA), proteomics (peptides), lipidomics (lipids), and so forth. The fundamental aspects of early visual staining and immunohistochemistry have persisted in areas that allow them to cater for these molecules. Shikotra et al. demonstrated the potential influence of thymic stromal lymphopoietin (TSLP) in severe asthma via the immunostaining of antigens within the mast cell axis [226]. In parallel, mass spectrometry techniques are now dominant for their capability of detecting molecular mass and facilitating untargeted investigations [227]. Mass spectrometry imaging (MSI) is a novel technique that aims to spatially integrate spectral analysis so that distinct molecules and their associated abundance can be mapped across a tissue section [228]. There are a few ion acquisition methods that facilitate MS imaging, each with their own degree of versatility, advantages and disadvantages.

### 4.12 Matrix‐Assisted Laser Desorption/Ionisation (MALDI)

The most widely documented and versatile method is matrix‐assisted laser desorption/ionisation or MALDI, which was born out of the work of Karas and Hillenkamp in their efforts to spatially represent Ca^2+^ ions in cardiac muscle cells [229, 230]. The logistics of MALDI involve a UV laser to spot‐desorb analytes and generate an ion plume to be analysed. An added organic matrix is theorised to absorb the laser energy and transfer it to the analyte, facilitating desorption and ionisation as well as protecting samples against fragmentation [231]. Sample preparation for MALDI imaging differs depending on the ome investigated and the tissue preservation method (fresh frozen, Formalin Fixed Paraffin Embedded (FFPE), or Formalin Fixed (FF)).

### 4.13 Desorption Electrospray Ionisation (DESI)

Desorption electrospray ionisation uses a charged solvent impacting upon a surface to ablate and ionise analytes without the need for a matrix and involving very minimal sample preparation [169]. For this reason, it is considered advantageous to MALDI, especially in the analysis of lipids and metabolites as they normally present close to MALDI matrix peaks. Lipid and metabolite analysis involves no sample preparation in DESI as most tissue samples intended for lipids and/or metabolomics analysis are fresh frozen.

Formalin‐fixed paraffin embedding is arguably the most popular embedding medium in modern histology as formalin fixation preserves tissue structure and paraffin infiltration allows samples to be kept at room temperature for decades [232]. The xylene wash required to remove paraffin during sample preparation also removes lipids and metabolites, leaving only the possibility for proteomics. Alternatively, carboxymethyl cellulose (CMC) has shown promise as a water‐soluble embedding medium to avoid this problem [233, 234]. Tissue sample preservation methods can restrict possible multi‐omic investigations due to the downstream protocols required to remove preservation material. In the absence of any embedding media, tissue may be fresh‐frozen short‐term to allow for all means of ‐omics analysis, however the lack of fixation means tissue structure is prone to decay and improvised desiccation procedures are needed to reduce moisture presence in favour of minimising analyte delocalisation. Optimal cutting temperature compound (OCT) is used to embed fresh‐frozen tissue onto a mount during cryostat sectioning, however steps are required to remove any residue as it is a known MS contaminant [234]. In addition, a graded ethanol wash series, common to all peptide and N‐Glycan analyses [235], removes residual lipids, salts and metabolites from the sample [236, 237]. The final proteomics‐exclusive step is an enzymatic digest (traditionally trypsin) to convert proteins into their constituent peptides [238].

### 4.14 Sample Preparation Considerations for Mass Spectrometry Imaging

There are several specific sample preparation considerations for both MALDI and DESI MSI. These arise from their hybrid nature as a combination of both MS and histology. Thus, the specific needs of both techniques must be accounted for, simultaneously, during sample preparation.

Sample preparation for MALDI‐ and DESI‐MSI begins with the embedding, which is when the tissue is enclosed in a mass of the embedding material using a mould. Traditional paraffin embedding is acceptable although should be avoided if possible (see FFPE section). The use of other compounds, including optimal cutting temperature (OCT) compound, should be avoided as they are preferentially ionised in the MS [239]. If used and not removed prior to acquisition, they can cause general ion suppression and significantly contaminate the instrument, sample losses and instrument down time. One exception to the above is that, when preparing frozen tissues, OCT can be used as an adhesive mount for the tissue block so long as it is not spread on the cutting face or blade of the cryotome [234]. If OCT use cannot be avoided, there are protocols to remove it prior to analysis [239], however these will remove any molecules soluble in water and hence prevent their analysis for multi‐omics. If samples are formalin fixed and not frozen, an alternative to OCT is carboxymethylcellulose and because it is inert, it will not ionise. Samples can then be sectioned using well‐established protocols [240].

Sectioning for MSI is performed at a larger thickness of 7–10 µm. The same considerations should be made for MSI as when performing histological staining and microscopy. Indeed, the artefacts that may affect the visual quality of a stained section should be removed so that the unevenness in the topography of the section does not affect the mass accuracy of the measurement taken with instruments that do not utilise an orthogonal acceleration in their ion path, such as TOF/TOF platforms [241]. Sections should be of even thickness. This is not a consideration in DESI, as it is most often hyphenated to quadrupole‐TOF instruments, but should still be avoided. Furthermore, it is advisable to wash microtome knives with acetone to remove any mineral oils and other contaminants that will suppress ionisation. Sectioned samples must be correctly mounted to indium tin oxide (ITO) coated glass slides for MALDI‐MSI (these are not required for DESI). These slides have a conductive coating allowing electricity to be passed through them, which greatly assists with ionisation by MALDI. Adhesive substrates can be used on the ITO slide, to ensure that tissues remain attached. However, a thin coating of liquid nitrocellulose (NC) offers the best results [242]. The need for strong adherence to the slide is due to the ultra‐high vacuum inside the MS. This can cause improperly adhered tissue to detach from the slide surface. Using thin coated NC can eliminate flaking and peeling.

After mounting, samples are prepared for either protein digestion or can be simply skipped to matrix coating if lipids and metabolites are desired. Fresh frozen tissues can be simply washed with a graded alcohol series to remove contaminating lipids. However for FFPE tissues, paraffin and formalin again need to be removed for the reasons stated previously [243]. Antigen retrieval is essential and there are numerous methods that unmask antigenic epitopes on FFPE tissues [244, 245, 246], which is covered extensively above. For multi‐omics workflows, it has been reported that a sequential analysis of lipids, metabolites and then peptides can be achieved in the same sample, however with each subsequent analysis the analyte abundance and intensity is reduced [247], this suggests that for the best multi‐omics results sequential “serial” sections should be used.

Tryptic digestion for MSI is conducted differently to standard proteomic workflows. Since the locations of the peptides inferring the ORF products are the required information, care must be taken not to delocalise the intact proteoforms through addition of the water‐containing environment required for proteolytic digestion to take place. There are varying approaches that can be taken for the application of trypsin [248, 249] (or any other enzyme), however, the most successful approach is to apply a concentrated solution of trypsin in deionised water to the surface of the tissue [105]. This should either be applied via a spraying apparatus (e.g., manual hobby sprayer) or by pipetting. The solution is allowed to dry without the slide being moved so that the trypsin molecules are deposited directly in contact with the surface proteoforms of the sample [105], minimising solubilisation of proteoforms and maintaining their spatial position and hence preventing delocalisation. The next step involves the activation of trypsin by creating the correct basic aqueous environment and temperature. Again, some methods choose to spray on a basic solution such as ammonium bicarbonate at pH 9.0 followed by incubation at 37°C [250]. However, samples can also be incubated at 37°C in a humidity chamber with a humid atmosphere of pH 9.0 [105]. This prevents the formation of surface droplets often seen with sprayers and minimises the delocalisation of proteoforms and the created peptides. Thus, the same effect as spraying or spreading is achieved, but in a more reproducible and standardised fashion [251].

The final stage of sample preparation for MALDI‐MSI is the coating of slides in an organic acid matrix needed to generate ions that can be measured by MS [252]. Matrix application has the same risks of sample delocalisation and is generally performed in one of two ways. The first is to apply a solvent and matrix mix sprayed directly onto the surface of the tissue [253]. The risk with this is that the droplets may form on the surface of samples and the peptides in the diameter of the droplet may move from their original to another location through diffusion [253]. This movement limits effective imaging resolution since large areas will contain the same homogenous mixtures of peptides, and the spatial specificity of those peptides will be reduced. Spraying systems aim to overcome this by producing misted droplets of the smallest possible size and not saturating the sample surface. The second involves the sublimation of matrix crystals [254], considered a dry coating method due to there being no liquid phase during either sublimation or deposition [255]. While requiring some empirical optimisation, the result is more uniform as the coating of homogenous matrix crystals are not wet, so they do not delocalise surface molecules. A drawback, however, is the lack of mixing of the analyte with the matrix, requiring recrystallisation in a humid chamber to incorporate the peptides with the matrix after its application [256]. The choice of matrix and whether spraying or sublimation are chosen has the ability to elicit different species and subspecies of molecules, allowing for multi‐omics analyses [250]. However as stated above, performing these on a single tissue section is a challenge and often necessitates serial sections. Nevertheless, with several sequential sections it is possible to have a comprehensive multi‐omics dataset for a single sample.

Other techniques such as Deep Visual Proteomics (DVP) [257, 258] and micro‐scaffold assisted spatial proteomics (MASP) [259], visium/xenium spatial RNAseq [260], and ICPMS, which results in complete destruction of the sample [261], can also be included in the general field of spatial multi‐omics, however, these techniques are tailored for a single ome analysis and are incompatible with other downstream omic analyses. While still being possible to integrate them with approaches such as MALDI and DESI by using serial sections, the differences in sample preparation techniques and spatial resolution makes their integration infeasible. We therefore consider these techniques to be complementary to mass spectrometry imaging but challenging to perform from the same tissue section due to either the complete ablation of the sample or changing architecture of cellular arrangements with thicker sections. In DVP, AI‐assisted image analysis is applied to train a model that determines the boundaries of different cells and whether the cells are morphologically different, such as being cancerous or benign [258]. These ‘shapes’, which are often more than one cell depending on tissue section thickness, are then excised from a tissue section using Laser Capture Microdissection and deposited into a well plate for extraction of the analytes. Thus, there is the potential to perform sequential multi‐omics extractions as described for bulk samples as the ‘shape’ removed is deposited into a well for further processing, but all of the challenges with sample loss from manipulation of single cells remains. We are not aware of any reports of this at the time of publication. While a similar approach, MASP applies a grid to the tissue section when sampling and thus a single cell could be cut into different grid locations. Otherwise, similar to DVP, sequential multi‐omics extractions as described for bulk samples could be performed, as mentioned by the authors [262].

### 4.15 Spatial Transcriptomics

A number of methods are available to visualise the spatial arrangements of cellular transcriptomes, as thoroughly reviewed by Wang et al. [263].These technologies are either imaging‐ or sequencing‐based with the difference being in the approach to determine spatial location and abundance of specific RNA molecules across the section, with imaging using fluorescent tagging and sequencing adding barcodes at a specific location then using next‐gen sequencing to determine a gene's expression level. As with all technologies, the selection of a particular one must be carefully aligned with the aim of the experiment. From a multi‐omics perspective, many articles claiming to perform a multi‐omics analysis do not conform to our definition.

## 5 A Brief Comment on Bioinformatics

While this review is focused on sample preparation, it would be churlish of us to neglect the final stage of any multi‐omics workflow, that being data integration and multi‐omics bioinformatics. The accessibility of user‐friendly extraction kits and advancements in protocol automation technologies have significantly contributed to an exponential increase in data generation over the last two decades. High‐throughput processes have resulted in vast amounts of data meaning that analysis is no longer intuitive and requires highly specialised computational approaches and infrastructure [264]. This phenomenon is particularly evident in genomics and transcriptomics, where computer science and bioinformatics play critical roles in generating sequence data, creating databases, and in annotating and curating data. This necessitates the use of specific tools and customised pipelines and some familiarity with coding. Specialised software and online portals are frequently used in proteomics to enable protein identification, quantification and interaction network analysis, while studies of metabolomes again require a different set of tools and computational approaches. This review does not delve into these methods, however, we want to emphasise the significance of the emerging field of systems bioinformatics, which seeks to integrate data across various omics layers [265].

Data integration in multi‐omics involves combining data from different cellular components or mapping them against each other to gain a comprehensive understanding of biological systems. Obtaining data from a single omics layer offers limited understanding, even if some inferences can be made. For instance, a complete genome sequence alone is insufficient to accurately predict the full repertoire of proteoforms. Thus, integrating multiple omics data provides a more comprehensive view of cellular processes and interactions. The main challenges include data heterogeneity, arising from the varying characteristics and scales of different omics data. Missing data and the diverse ways of handling it also generate difficulties. Computational complexities increase as datasets grow larger or the more diverse the datasets being integrated, especially when wanting to integrate spatial and/or temporal aspects of each omics [56]. Studying the system as a whole rather than analysing its components in isolation, is the ultimate goal which cannot be achieved without the use of appropriate bioinformatic methods.

## 6 Conclusion and Deciding on the Appropriate Workflow

As mentioned above, the consideration of which extraction method to employ is a careful compromise between the amount of sample that can be used and the necessity of gaining multiple omics datasets from a single sample. As demonstrated, there are numerous compatibility and temporal considerations associated with many different types of analytes and the desire to analyse each ome at the best possible quality. We therefore conclude that the best possible approach for performing sample preparation destined for multi‐omics analysis is to first homogenise the raw sample then divide it up equally before running each fraction through the intended downstream analysis. If this is not possible, then the ome that is the most needed or desirable should be the primary focus for sample preparation.

## Conflicts of Interest

The authors declare no conflicts of interest.

## Data Availability

The authors have nothing to report.
